# Letter from the Editor in Chief

**DOI:** 10.19102/icrm.2023.14076

**Published:** 2023-07-15

**Authors:** Moussa Mansour


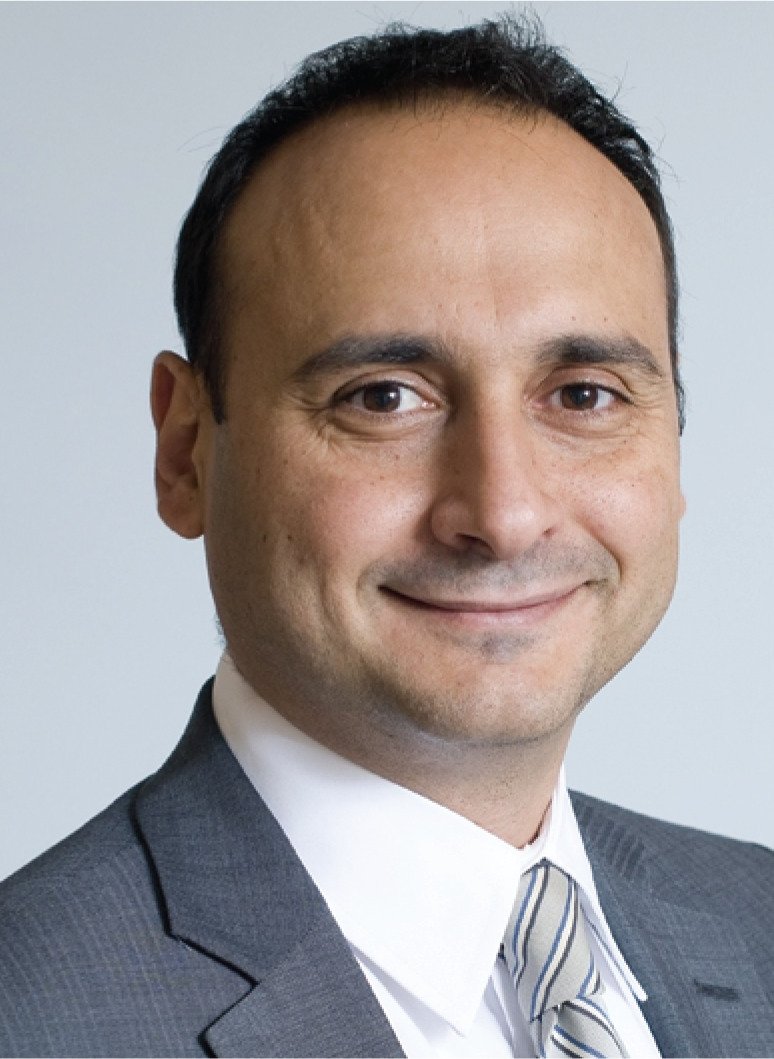


Dear readers,

In the field of ablation for atrial fibrillation (AF), high-power, short-duration (HPSD) delivery has been performed for the past several years. It gained popularity because it shortens the procedure time and improves the success rate, as demonstrated by numerous publications. This issue of *The Journal of Innovations in Cardiac Rhythm Management* contains a meta-analysis by Kumar et al. titled “Comparative Efficacy and Safety Profiles of High-power, Short-duration and Low-power, Long-duration Radiofrequency Ablation in Atrial Fibrillation: A Systematic Review and Meta-analysis” in which the authors reviewed 21 studies on this subject with a total of 4,169 patients.^1^ Their results confirmed the findings of prior studies, demonstrating the superiority of HPSD for ablation for AF—specifically, the use of this technique reduces the procedural time, number of ablation lesions, and fluoroscopy time. Moreover, HPSD also lessens the frequency of esophageal thermal injury, pulmonary venous reconnection, and AF recurrence.

While HPSD has become the main technique used for AF ablation, its definition remains unclear. Among the 21 studies described in the above-mentioned manuscript, there was significant variation in how high power was defined: some studies used 40 W of radiofrequency (RF) energy, while others used 70 W. Similarly, the definition of short duration varied from 2–10 s. These variations led to an inability to develop a consensus for defining HPSD, especially one that can be used on a widescale basis. One major reason for such variation was differences in measurement of the catheter–tissue interface. With conventional technology, the temperature sensor is located at the center of the catheter and, as a result, its ability to measure accurate temperature is affected by the surrounding irrigation fluid. This problem has been addressed in newer-generation RF catheters, which contain multiple temperature sensors inserted at the surface of the catheter, allowing for accurate measurements of the catheter–tissue interface. Accurate temperature measurement maximizes energy delivery; by setting an upper limit for temperature, maximum energy delivery is permitted and limited only by temperature. Safety also is improved because limiting temperature rises results in less char formation and fewer steam pops. Moreover, accurate temperature measurement also facilitates the development of ablation index formulas, which results in better lesion size estimation.

In summary, the widescale use of HPSD is supported by a large amount of data, and newer-generation RF catheters with multiple surface temperature sensors enable maximal energy delivery and fewer complications.

I hope that you enjoy reading the remaining articles in this issue of *The Journal of Innovations in Cardiac Rhythm Management*.


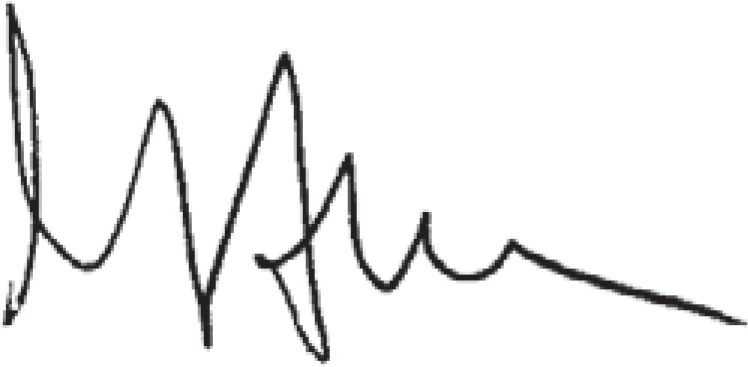


Sincerely,

Moussa Mansour, md, fhrs, facc

Editor in Chief

*The Journal of Innovations in Cardiac Rhythm Management*

MMansour@InnovationsInCRM.com

Director, Atrial Fibrillation Program

Jeremy Ruskin and Dan Starks Endowed Chair in Cardiology

Massachusetts General Hospital

Boston, MA 02114
